# Supplementary material for: DMRforPairs: identifying Differentially Methylated Regions between unique samples using array based methylation profiles
Source: BMC Bioinformatics. 2014 May 15;15:141. doi: 10.1186/1471-2105-15-141 (PMC4046028; doi:10.1186/1471-2105-15-141)
Supplement: Additional file 2 — DMRforPairs output for the comparison of A431-MCF7 and NA17018-NA17105. Please start from the HTML files in each folder. Available via the BMC Bioinformatics website. [file 1471-2105-15-141-S2.zip › 1394847754114233_MOESM2_ESM/A431_MCF7/figures/17302.pdf]

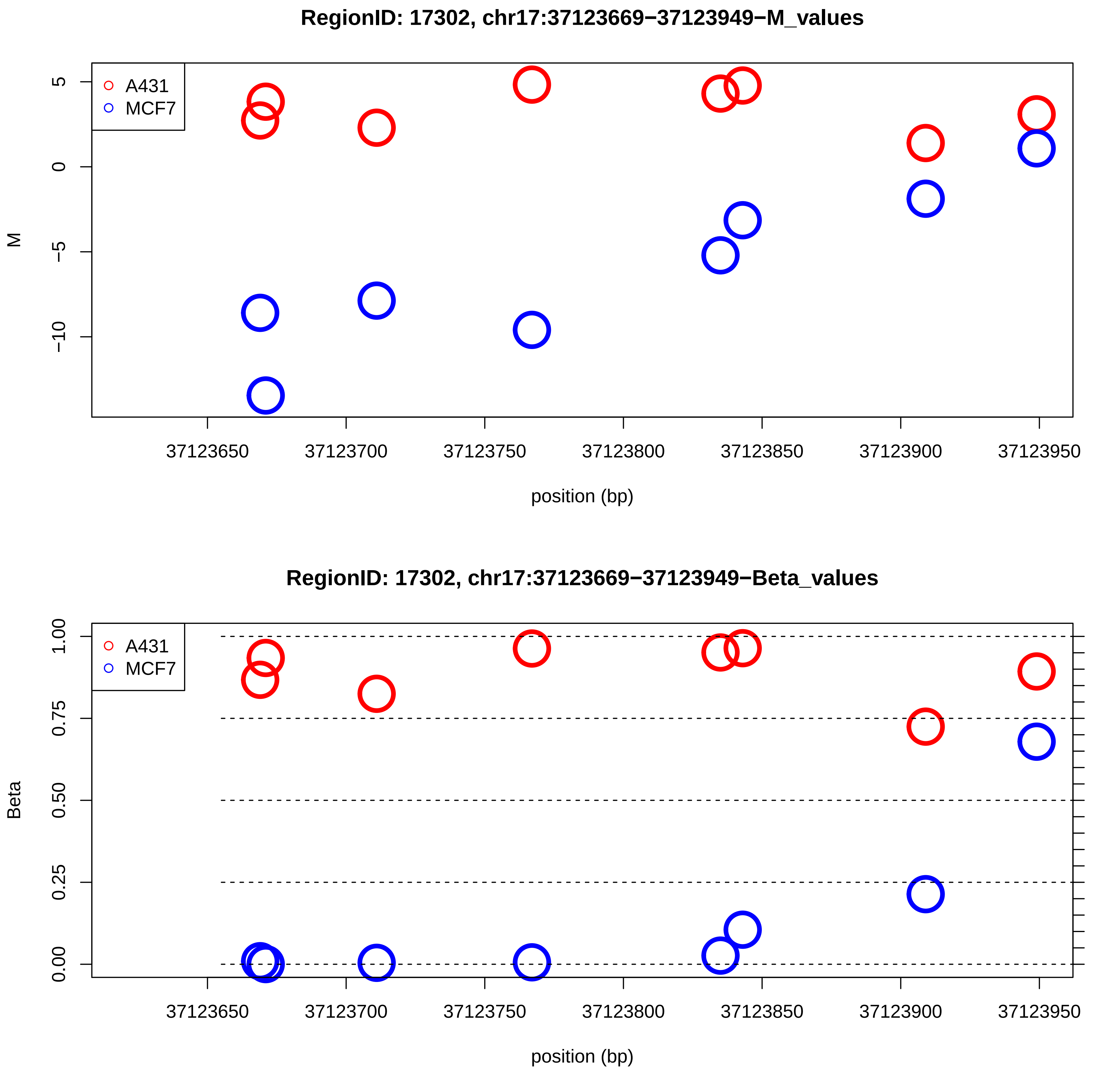

RegionID: 17302, chr17:37123669−37123949−M_values
A431
MCF7
M
5
0
−5
−10
37123650
37123700
37123750
37123800
37123850
37123900
37123950
position (bp)
RegionID: 17302, chr17:37123669−37123949−Beta_values
A431
MCF7
Beta
1.00
0.75
0.50
0.25
0.00
37123650
37123700
37123750
37123800
37123850
37123900
37123950
position (bp)

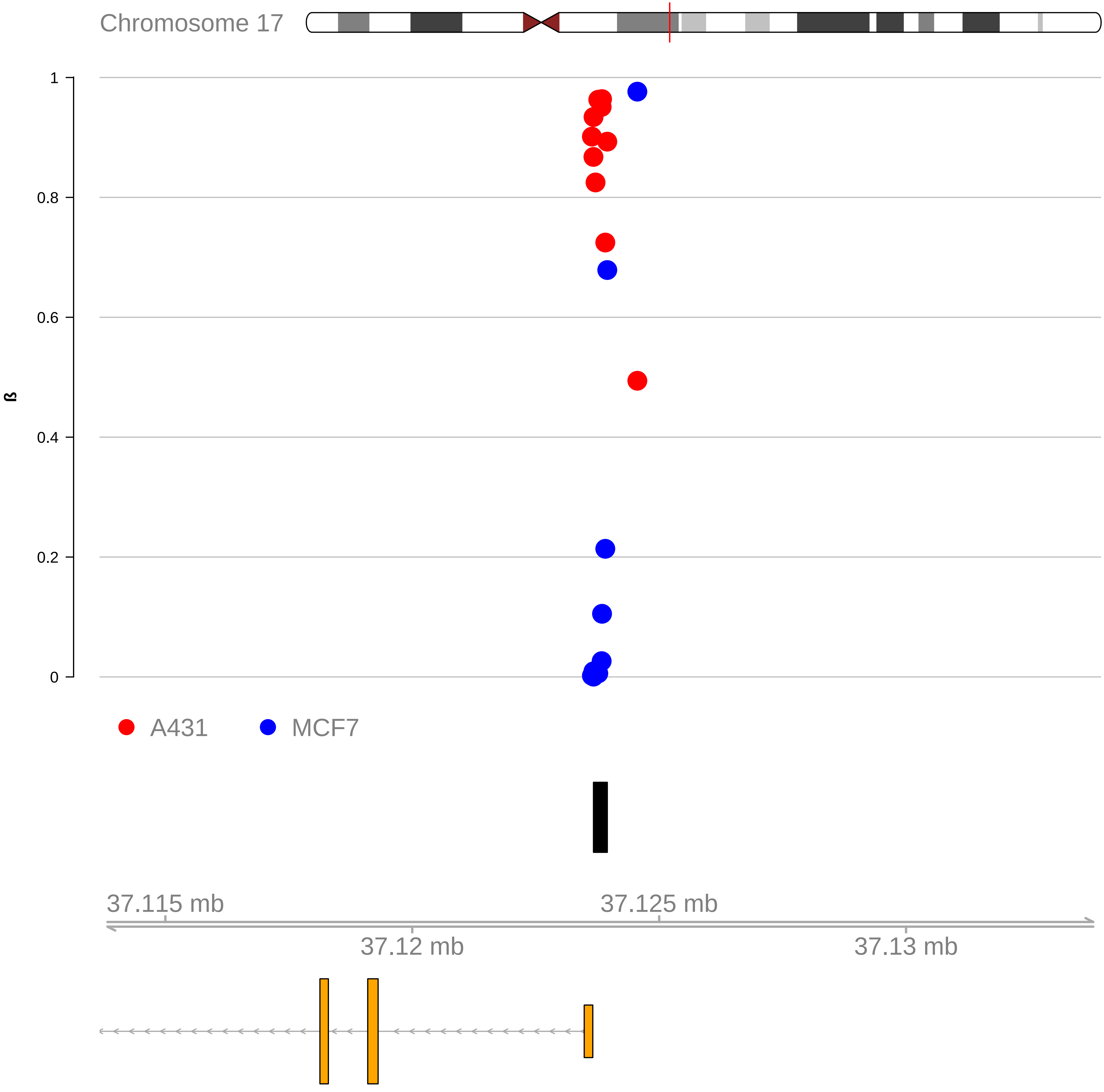

Chromosome 17
1
0.8
0.6
0.4
0.2
0
ß
A431
MCF7
37.115 mb
37.125 mb
37.12 mb
37.13 mb
